# Socioecologic Factors as Predictors of Readiness for Self-Management and Transition, Medication Adherence, and Health Care Utilization Among Adolescents and Young Adults With Chronic Kidney Disease

**DOI:** 10.5888/pcd11.140072

**Published:** 2014-07-10

**Authors:** Karina Javalkar, Nicole Fenton, Sarah Cohen, Maria Ferris

**Affiliations:** Author Affiliations: Karina Javalkar, Sarah Cohen, University of North Carolina–Chapel Hill, Chapel Hill, North Carolina; Nicole Fenton, Children’s Hospital of Philadelphia, Philadelphia, Pennsylvania.

## Abstract

**Introduction:**

The objective of our study was to determine the socioecologic factors that predict readiness for self-management and transition from pediatric to adult health care services, adherence to taking medications, and health care utilization among adolescents and young adults with chronic kidney disease.

**Methods:**

We enrolled 52 adolescents and young adults aged 13 to 21 (96.5% participation). Participants were administered measures that examined: socioecologic factors, individualized education plans or 504 plans, readiness for self-management and transition (the University of North Carolina TRxANSITION scale), triangulated measures of adherence to taking medications (parent reported, physician reported, and medication-possession ratios), and health care utilization (number of visits to the emergency department, number of inpatient admissions, and number of inpatient days in the previous year).

**Results:**

Overall, our sample had moderate levels of readiness for self-management and transition, high rates of parent- and physician-reported medication adherence, and high rates of health care utilization. Age was a significant positive predictor of readiness for self-management and transition. Compared with participants who had private health insurance, participants who had public insurance had more emergency department visits, inpatient admissions, and inpatient days, and lower rates of physician-reported medication adherence. Participants who did not have an individualized education plan or 504 plan had significantly more emergency department visits, inpatient admissions, and inpatient days.

**Conclusion:**

Socioecologic factors play an important role in readiness for self-management and transition, medication adherence, and health care utilization in pediatric patients with chronic kidney disease. Age, insurance status, and having an individualized education plan or 504 plan may be key factors.

## Introduction

Understanding the socioecologic factors that predict health outcomes in chronic disease may provide insight for designing effective interventions. Adolescents and young adults with chronic health conditions are vulnerable to health risks when they start to self-manage their condition. Inadequate preparation for the transition from pediatric- to adult-focused health care services may result in negative health outcomes (1). Disease self-management, adherence to taking medications, and health care utilization are 3 important outcomes during this transition process (1,2).

Few studies of youth with chronic conditions investigate how socioecologic factors predict outcomes. One study of children with chronic kidney disease (CKD) found that those with low perceived health status had higher rates of health care utilization (3). In another study, children and adults with Medicaid insurance were more likely to visit the emergency department than those with commercial insurance (4).

The objective of our study was to determine the socioecologic factors that predict readiness for self-management and transition from pediatric to adult health care services, adherence to taking medications, and health care utilization among adolescents and young adults with CKD.

## Methods

### Data collection

We recruited a convenience sample in the clinic at the University of North Carolina (UNC) Kidney Center in Chapel Hill, North Carolina, during 2012 and 2013. To be eligible to participate, patients had to be aged from 13 to 21 years and have a diagnosis of CKD (stage 2 or above) for more than 3 months. Potential participants who did not speak English fluently (or their parents if the patients were younger than 18) were excluded from the study, as were those who had significant cognitive or developmental delays. Eligibility was determined on the basis of the health provider’s assessment and a review of the electronic medical record (5).

The study was approved by the institutional review board at UNC–Chapel Hill. Once eligible participants were in their clinic rooms, they were approached by a trained research assistant and provided with a study overview. If the potential participant expressed a desire to participate, informed assent and consent forms and Health Insurance Portability and Accountability Act forms were completed by the participants or their guardians. Participants first completed the 33-item UNC TRxANSITION scale (6) administered by the research assistant. They then answered questions on socioecologic factors, health care utilization, and medication adherence by using the Web-based survey engine Qualtrics (Qualtrics, LLC, Provo, Utah). If the participants did not have time to complete the Web-based questionnaires at the clinic, they were given a Web link to access the survey on their home computers. Also during the clinic visit, parents were asked to rate their child’s adherence to taking medications. On completing the study visit, physicians were asked to rate the adherence of the participant, and pharmacies were contacted to obtain medication refill records.

### Measures

**Readiness for self-management and transition.** The UNC TRxANSITION scale was used to assess disease self-management and the participant’s readiness to transition from pediatric to adult health care (6). This survey is administered in person by a health care provider or trained research assistant. It assesses disease self-efficacy (what the person thinks they can do to manage their illness) and disease knowledge as continuous variables. The scale has 10 subscales: 1) knowledge of type of illness, 2) knowledge of prescriptions, 3) adherence to treatment, 4) knowledge of nutrition restrictions, 5) self-management skills, 6) knowledge of reproduction issues, 7) knowledge of importance of a trade or school (knowledge of participants’ plans for school or a job), 8) knowledge of insurance, 9) identification of ongoing support (knowledge of how to take care of themselves when they become adults), and 10) knowledge of how to find new [health care] providers. Each question in the scale is scored by the administrator as follows: 0 points (no knowledge or self-management), 0.5 points (some knowledge or self-management), or 1 point (complete knowledge or self-management). A total score is calculated (range, 0–10) and reflects overall readiness for health care transition. Adequate reliability has been found (κ = 0.70), and other information on validity has been published (6).

**Socioecologic factors.** A self-report, Web-based questionnaire was created for this study. We asked participants about their age, race/ethnicity (non-Hispanic white, African American, Hispanic, Native American, Asian, and “other”), sex (male, female), use of an individualized education plan (IEP) or 504 plan (yes or no), insurance status (private, public [Medicare or Medicaid], or self-pay), age at diagnosis of CKD, and parents’ highest grade of school completed. An IEP is a plan or program in which a child who has a disability identified under the law and is attending an elementary or secondary educational institution receives specialized instruction and related services. A 504 plan ensures that such a child receives appropriate accommodations. We collected data on IEP and 504 plans only from participants who had not yet graduated from high school (n = 40). Data on the number of prescribed medications were obtained from the medical record.

**Health care utilization.** We asked participants to report the number of times in the previous year they had visited the emergency department, the number of times they had been admitted to the hospital, and the total number of days they had spent as a hospital inpatient. These questions were administered online, and answers were treated as continuous variables.

**Medication adherence.** Medication adherence was estimated as a continuous variable by using 3 sources: parent report, physician report, and pharmacy refill records; the latter were used to calculate the medication-possession ratio (MPR). Each participant’s parent and subspecialist were asked (in person) to estimate (confidentially) the percentage of time they think the participant does not take his or her medications. The questions were “Please rate on a scale of 0 to 100 how adherent you think the following patient is to their medications” and “Please rate on a scale of 0 to 100 how adherent you think your child is to their medications (how often do they take their medications as prescribed?).” The response range was 0 to 100, with 0 being that the participant never took the medications and 100 being that the participant adhered perfectly. This question was modeled after studies that used 1 question to estimate medication adherence (7–9).

The MPR was used as an objective measure of adherence. We obtained each participant’s pharmacy refill records for 1 year before the date of study participation and used it to calculate the 3-month MPR. Studies have found that the MPR for a 3-month period is representative of a patient’s typical adherence (10). The pharmacy refill record included a list of medications and the date each was filled or refilled at that pharmacy. We defined MPR as the proportion of prescribed medications obtained by the participant during a specific period [MPR = (number of days the participant had a supply of the medication during the observation period ÷ number of days in observation period) × 100]. This method is consistent with previous studies (9,10).

### Statistical analysis

A power analysis was calculated by using SAS 9.3 TS1M2 (SAS Institute Inc, Cary, North Carolina). Assuming a moderate effect size (0.4) and a 1-tailed α of .05, we determined that a sample size of 42 would be sufficient to obtain power of .80 for our model. We calculated the participation rate as the number of patients that agreed to participate divided by the number of eligible patients approached. We calculated the percentage of life that the participant had CKD by subtracting age at diagnosis from current age and then dividing by current age. Regression analyses were conducted to examine the socioecologic factors that predict readiness for self-management and transition, medication adherence, and health care utilization. The factors in each of the regression analyses were the following independent variables: age, sex, race/ethnicity, insurance status, percentage of life with disease; use of an IEP or 504 plan, and number of prescribed medicines. For the regression analyses on self-management and transition readiness, we used the total TRxANSITION score as well as the scores for each of the subscales as the outcomes of interest. Significance was determined at P ≤ .05.

Descriptive statistics were calculated and linear regression analyses were conducted by using IBM SPSS Statistics 22 (IBM Corporation, Armonk, New York).

## Results

We enrolled 52 participants (96.5% participation): 30 (58%) were male; 16 (30%) were non-Hispanic white, and 26 (50%) were African American; the average age was 17.6 years (range, 13–21 y) (Table 1). Thirty-one percent had private insurance, 62% had public insurance, and 8% were self-pay. On average, participants had lived with their disease for 46.4% (standard deviation [SD], 35.8%) of their life. On average, participants were prescribed 4.7 (SD, 2.7) medications, and 21 of 40 (52.5%) participants who answered the question about having an IEP or 504 plan had one.

**Table 1:** Characteristics of Participants (n = 52) in Study of Adolescents and Young Adults With Chronic Kidney Disease, Chapel Hill, North Carolina

Characteristic	Value^a^
**Race/ethnicity**	
Non-Hispanic white	16 (31)
African American	26 (50)
Hispanic	1 (2)
Native American	6 (12)
Asian	1 (2)
Other	2 (4)
**Sex**	
Male	30 (58)
Female	22 (42)
**Age, mean (SD), y**	17.6 (2.2)
**Percentage of life with disease, mean (SD)**	46.4 (35.8)
**Insurance status**	
Private insurance	16 (31)
Public insurance	32 (62)
Self-pay	4 (8)
**No. of medications, mean (SD)**	4.7 (2.7)
**Has an IEP/504 plan^b^**	21 (52.5)
**Highest school grade attained by parent, mean (SD)**	13.6 (2.5)

Abbreviations: SD, standard deviation: IEP, individualized education plan.

a Values are mean (%) unless otherwise indicated. Percentages may not sum to 100% because of rounding.

b An IEP is a plan or program in which a child who has a disability identified under the law and is attending an elementary or secondary educational institution receives specialized instruction and related services. A 504 plan ensures that such a child receives appropriate accommodations. We collected data on IEP and 504 plans only from participants who had not yet graduated from high school (n = 40).

The mean total score for the TRxANSITION scale was 6.9 (SD, 1.4) (Table 2). The rate of health care utilization was high; on average, participants visited the emergency department 3.4 (SD, 8.6) times and had 3.1 (SD, 7.9) hospital admissions in the previous year. According to parent and physician reports, the study group had a high rate of medication adherence. The average rate reported by parents was 85.5% (SD, 22.8%), and the average rate reported by physicians was 80.7% (SD, 21.2%). However, the MPR showed that participants had a supply of their medications only 55.0% (SD, 50.4%) of the time.

**Table 2:** Readiness for Self-Management and Transition, Medication Adherence, and Health Care Utilization by Adolescents and Young Adults With Chronic Kidney Disease (N = 52), Chapel Hill, North Carolina

Outcome	Mean (Standard Deviation)
**Total TRxANSITION^a^ score**	6.9 (1.4)
Know type of illness	0.74 (0.26)
Know Rx (prescriptions)	0.71 (0.22)
Be adherent to treatment	0.82 (0.23)
Know nutrition restrictions	0.64 (0.31)
Have self-management skills	0.54 (0.30)
Know issues of reproduction	0.61 (0.32)
Know the importance of a trade or school	0.81 (0.33)
Know about insurance	0.56 (0.28)
Identify ongoing support	0.91 (0.19)
Know how to find new providers	0.57 (0.31)
**Health care utilization**	
No. of emergency department visits in previous year	3.4 (8.6)
No. of inpatient admissions in previous year	3.1 (7.9)
No. of total inpatient days in previous year	5.6 (9.0)
**Adherence (range, 0–100)**	
Medication-possession ratio^b^	55.0 (50.4)
Physician rating of medication adherence	80.7 (21.2)
Parent rating of medication adherence	85.5 (22.8)

a The 33-item University of North Carolina TRxANSITION scale ranges from 0 to 10.

b Defined as the proportion of prescribed medications obtained by the participant during a specific period [MPR = (number of days the participant had a supply of the medication during the observation period ÷ number of days in observation period) × 100].

Age was a significant positive predictor of the total TRxANSITION score (β = 0.384; *P* = .046) and the disease self-management subscale (β = 0.361; *P* = .04) (Table 3). Sex was a significant predictor of one TRxANSITION outcome: female participants had higher scores for the trade or school subscale (β = 0.431; *P* = .03). Participants with private insurance had greater knowledge of who would manage their illness when they became adults (ongoing support subscale, β = −0.422; *P* = .05).

**Table 3:** Predictors of Readiness for Self-Management and Transition and Health Care Utilization Among Adolescents and Young Adults With Chronic Kidney Disease, Chapel Hill, North Carolina

Variable	β	*P* Value for Linear Regression
**Total TRxANSITION score**		
Insurance^a^	−0.128	.52
Age	0.384	.05
Percentage of life with disease	−0.151	.45
Sex^b^	0.305	.10
Race^c^	0.027	.90
IEP/504 plan^d^	−0.162	.41
Total no. of prescribed medicines	0.037	.84
**TRxANSITION disease self-management**		
Insurance^a^	0.081	.66
Age	0.361	.04
Percentage of life with disease	0.154	.41
Sex^b^	0.072	.67
Race^c^	0.261	.21
IEP/504 plan^d^	−0.263	.16
Total no. of prescribed medicines	0.070	.69
**TRxANSITION trade or school**		
Insurance^a^	−0.193	.34
Age	0.023	.90
Percentage of life with disease	−0.115	.57
Sex^b^	0.431	.03
Race^c^	−0.196	.39
IEP/504 plan^d^	−0.071	.72
Total no. of prescribed medicines	0.018	.93
**TRxANSITION ongoing support**		
Insurance^a^	−0.422	.05
Age	−0.158	.41
Percentage of life with disease	−0.126	.54
Sex^b^	−0.185	.34
Race^c^	0.208	.37
IEP/504 plan^d^	0.008	.97
Total no. of prescribed medicines	0.159	.43
**No. of emergency department visits in previous year**		
Insurance^a^	0.423	.02
Age	0.672	.001
Percentage of life with disease	0.000	>.99
Sex^b^	0.353	.04
Race^c^	−0.669	.004
IEP/504 plan^d^	0.369	.04
Total no. of prescribed medicines	0.041	.82
**No. of hospital admissions in previous year**		
Insurance^a^	0.405	.04
Age	0.675	.001
Percentage of life with disease	0.002	.99
Sex^b^	0.312	.07
Race^c^	−0.601	.01
IEP/504 plan^d^	0.429	.03
Total no. of prescribed medicines	0.008	.96
**Total no. of inpatient days in previous year**		
Insurance^a^	0.360	.01
Age	0.751	<.001
Percentage of life with disease	0.055	.70
Sex^b^	0.325	.02
Race^c^	−0.623	.001
IEP/504 plan^d^	0.447	.003
Total no. of prescribed medicines	−0.093	.51
**Physician rating of medication adherence**		
Insurance^a^	−0.727	.009
Age	0.033	.86
Percentage of life with disease	−0.196	.32
Sex^b^	−0.024	.89
Race^c^	0.262	.33
IEP/504 plan^d^	0.215	.26
Total no. of prescribed medicines	0.010	.96

Abbreviations: IEP, individualized education plan.

a A positive value for β indicates public insurance or self-pay as a predictor, whereas a negative value indicates private insurance.

b A positive value for β indicates that female sex is a predictor, whereas a negative value indicates male sex.

c A negative value for β indicates the white race as a predictor, whereas a positive value indicates another race (African American, Hispanic, Native American, Asian, or “other”).

d A positive value for β indicates not having an IEP or 504 plan, whereas a negative value indicates having one.

Having public insurance, increasing age, being female, being white, or not having an IEP or 504 plan were each significant predictors of greater rates of health care utilization (Table 3). Participants who had public insurance (β = 0.423; *P* = .02), who were older (β = 0.672; *P* = .001), were female (β = 0.353; *P* = .04), were white (β = −0.669; *P* = .004), or did not have an IEP or 504 plan (β = 0.369, *P* = .04) had more emergency department visits in the previous year. These same factors also predicted more days spent as an inpatient in the previous year (Table 2). Similarly, participants who had public insurance (β = 0.405; *P* = .04), were older (β = 0.675; *P* = .001), were white (β = −0.601; *P* = .01), or did not have an IEP or 504 plan (β = 0.429; *P* = .03) had more hospital admissions in the previous year. The overall models predicting emergency departments visits (*P* = .005), hospital admissions (*P* = .01), and total number of inpatient days (*P* < .001) suggested that socioecologic factors were significant predictors of health care utilization outcomes.

None of the socioecologic factors predicted the rates of medication adherence indicated by parents or the MPRs. However, insurance status was a significant predictor of physician-reported medication adherence. Participants with private insurance received a higher physician-reported adherence rating (β = −0.727, *P* = .009) than participants with public or self-pay insurance.

## Discussion

This study examined the role of socioecologic factors in predicting readiness for self-management and health care transition, medication adherence, and health care utilization among adolescents and young adults with CKD. The study included a large proportion of racial/ethnic minorities, which was representative of the CKD population served at the UNC Kidney Center and the population of CKD patients served across the country (11).

In our study, participants who had private insurance had significantly higher rates of physician-reported medication adherence and significantly greater knowledge of how to take care of themselves when they became adults than participants with public insurance. Health care utilization outcomes were also predicted by insurance status. Participants who had private insurance spent fewer total days admitted in the hospital than those with public insurance. Our results are consistent with those of previous studies, which showed that pediatric patients with public health insurance may have higher rates of emergency department visits than those with private insurance (12). Public insurance therefore appears to be an important factor for poor transition readiness and negative health care utilization outcomes.

We found that having IEPs or 504 plans were significant predictors of the outcomes examined. Participants that had an IEP or 504 plan had significantly fewer emergency department visits, fewer number of times they were admitted to the hospital, and fewer number of inpatient days in the previous year, suggesting that their IEP or 504 plan may be aiding in keeping them healthy and away from the emergency department or a hospitalization. An IEP or 504 plan was generally a predictor of positive outcomes in this population. Perhaps the school accommodations help to minimize stress, which increases immune functioning and results in lower rates of emergency health care utilization (13). Factors that account for this relationship should be explored in future investigations. It may be important to ensure that those with CKD who need an IEP or 504 plan receive one; having one may put them at less risk for negative health care utilization outcomes.

Our study has several limitations. First, the measures of health care utilization were self-reported. Second, we were unable to control for disease severity directly (although we did control for percentage of life with disease and number of prescribed medications). There may be other potentially confounding variables that were not explored in this study. Third, 2 of the 3 measures of medication adherence (parent reported and physician reported) may have underlying biases. To overcome this limitation, we made every effort to use all 3 measures of adherence, including the objective measure, the MPR. Fourth, this was a single-center study. We are expanding our observations to other institutions to increase the generalizability of our findings.

Socioecologic variables are often used as control variables when trying to understand a patient’s level of adjustment to a chronic illness. This approach often results in the explanatory role of demographic variables themselves being overlooked. We found that these socioecologic factors allowed for a partial understanding of readiness for self-management and transition, medication adherence, and health care utilization in pediatric patients with CKD. Socioecologic factors were an especially helpful way to conceptualize data on the number of emergency visits and hospitalizations. Important predictors of readiness for self-management and transition to self-management among adolescents and young adults with CKD included age, insurance status, and having an IEP or 504 plan.

## References

[1] Watson AR . Problems and pitfalls of transition from paediatric to adult renal care. Pediatr Nephrol 2005;20(2):113–7. 10.1007/s00467-004-1763-y 15627164

[2] Ferris ME , Mahan JD . Pediatric chronic kidney disease and the process of health care transition. Semin Nephrol 2009;29(4):435–44. 10.1016/j.semnephrol.2009.03.018 19615564

[3] Gerson AC , Riley A , Fivush BA , Pham N , Fiorenza J , Robertson J , Assessing health status and health care utilization in adolescents with chronic kidney disease. J Am Soc Nephrol 2005;16(5):1427–32. 10.1681/ASN.2004040258 15772253

[4] Kappelman MD , Porter CQ , Galanko JA , Rifas-Shiman SL , Ollendorf DA , Sandler RS , Utilization of healthcare resources by U.S. children and adults with inflammatory bowel disease. Inflamm Bowel Dis 2011;17(1):62–8. 10.1002/ibd.21371 20564532 PMC2962765

[5] National Kidney Foundation. Clinical practice guidelines for chronic kidney disease: evaluation, classification and stratification. K/DOQI clinical practice guidelines. Am J Kidney Dis 2002;39(2 Suppl 1):S1–266. 10.1016/S0272-6386(02)70054-1 11904577

[6] Ferris ME , Bickford K , Layton JB , Ferris T , Ford C , Hogan S , A clinical tool to measure the components of health-care transition from pediatric care to adult care: the UNC TR(x)ANSITION scale. Ren Fail 2012;34(6):744–53. 10.3109/0886022X.2012.678171 22583152

[7] Matthews JR , Christopherson ER . Measuring and preventing noncompliance in pediatric health care. In: Karoly P, editor. Handbook of child health assessment. New York (NY): John Wiley & Sons; 1988. p. 519–57.

[8] La Greca AM . Adherence to prescribed medical regimens. In: Routh DK, editor. Handbook of pediatric psychology. New York (NY): The Guilford Press; 1988. p. 299–320.

[9] Cluss PA , Epstein L . The measurement of medical compliance in the treatment of disease. In: Karoly P, editor. Measurement strategies in health psychology. New York (NY): John Wiley & Sons; 1985. p. 403–32.

[10] Andrade SE , Kahler KH , Frech F , Chan KA . Methods for evaluation of medication adherence and persistence using automated databases. Pharmacoepidemiol Drug Saf 2006;15(8):565–74. 16514590 10.1002/pds.1230

[11] Harambat J , Stralen K , Kim J , Tizard E . Epidemiology of chronic kidney disease in children. Pediatr Nephrol 2012;27(3):363–73. 10.1007/s00467-011-1939-1 21713524 PMC3264851

[12] Fong C . The influence of insurance status on nonurgent pediatric visits to the emergency department. Acad Emerg Med 1999;6(7):744–8. 10.1111/j.1553-2712.1999.tb00446.x 10433536

[13] Segerstrom SC , Miller G . Psychological stress and the human immune system: a meta-analytic study of 30 years of inquiry. Psychol Bull 2004;130(4):601–30. 10.1037/0033-2909.130.4.601 15250815 PMC1361287

